# Therapeutic Potential of Proanthocyanidins in Ulcerative Colitis in Remission

**DOI:** 10.3390/jcm9030771

**Published:** 2020-03-12

**Authors:** Dominika Głąbska, Dominika Guzek, Karolina Gałązka, Gustaw Lech

**Affiliations:** 1 Department of Dietetics, Institute of Human Nutrition Sciences, Warsaw University of Life Sciences (SGGW-WULS), 159C Nowoursynowska Street, 02-787 Warsaw, Poland; karolina.galazka11@gmail.com; 2 Department of Food Market and Consumer Research, Institute of Human Nutrition Sciences, Warsaw University of Life Sciences (SGGW-WULS), 159C Nowoursynowska Street, 02-787 Warsaw, Poland; dominika_guzek@sggw.pl; 3 Department of General, Gastroenterological and Oncological Surgery, Medical University of Warsaw, 1a Banacha Str., 02-097 Warsaw, Poland; gustaw.lech@wum.edu.pl

**Keywords:** ulcerative colitis, remission, proanthocyanidins, dietary intake, symptoms

## Abstract

A number of bioactive components of diet are indicated as potential dietary factors for the management of ulcerative colitis, while the recent study conducted in an animal model revealed that proanthocyanidins from grape seeds exert a broadly positive impact. The aim of the study was to verify the influence of dietary proanthocyanidins intake on the symptoms of ulcerative colitis in remission in human subjects. The study was conducted in a group of 55 participants (19 males, 36 females) in remission of ulcerative colitis confirmed based on both the Mayo Scoring system and Rachmilewitz index. Their habitual dietary intake of proanthocyanidins and intake recalculated per 1000 kcal of diet was assessed and it was verified whether they are associated with symptoms of ulcerative colitis. The energy value of diet and intake of other nutrients were analyzed as potential interfering factors. Participants declaring the presence of mucus in their stool compared with other participants were characterized by higher proanthocyanidins intake (142 vs. 75 mg; *p* = 0.0441) and intake per 1000 kcal (91 vs. 37 mg/1000 kcal; *p* = 0.0092), while for no other nutrient such association was stated. Participants declaring constipation compared with other participants were characterized by higher proanthocyanidins intake (214 vs. 82 mg; *p* = 0.0289) and intake per 1000 kcal (118 vs. 41 mg/1000 kcal; *p* = 0.0194). Similar association for constipation was observed in the case of energy value of diet and protein intake, but only for proanthocyanidins intake, it was confirmed in the logistic regression model (*p* = 0.0183; OR = 1.01; 95% CI 1.00–1.02). The positive influence of habitual dietary intake of proanthocyanidins was confirmed in the studied group of patients with ulcerative colitis in remission, as this intake may have increased the production of mucus, which is beneficial for intestinal healing, and may have reduced the frequency of bowel movements. However, further experimental human studies are necessary to confirm the potential influence of proanthocyanidins intake in patients with ulcerative colitis in remission.

## 1. Introduction

Ulcerative colitis is classified as one of the inflammatory bowel diseases, defined as a chronic inflammatory condition resulting in diffuse friability and superficial erosions of the colonic wall, associated with bleeding [1]. Alternating periods of remission and relapse are the typical elements of the course of ulcerative colitis, while relapses are commonly difficult to predict [2]. At the same time, there are numerous symptoms of this disease, including abdominal pain, diarrhea, or rectal bleeding [3], which may be experienced even in remission [4] and may reduce the general quality of life of patients [5]. Considering these aspects, patients with ulcerative colitis need adequate care during both relapses and remissions [6].

Proper nutrition is one of the most important issues that should be addressed in patients with ulcerative colitis [7]. This is because nutrition is not only involved in pathogenesis but also may play a role in the management of the disease [8]. However, for the period of remission, there are no defined dietary guidelines associated with a specific nutritional value of diet, but rather more general recommendations associated with food products that should be avoided, reduced, or increased in the everyday diet [9].

Many bioactive components of diet are indicated as potential dietary factors for the management of ulcerative colitis, and these components are even thought to be possible alternative therapeutic agents in the future [10]. These bioactive components have a wide spectrum of positive health effects that have already been proven in in vitro studies and animal model studies, and depending on the component, they may be involved in antioxidant defenses, cell proliferation, and gene expression—all these processes being important for the course of ulcerative colitis [11].

Flavonoid compounds are one of the potentially beneficial components that may influence the course of ulcerative colitis. Flavonoids are plant-derived hydroxylated polyphenolic molecules that are characterized mainly by antioxidant activity, together with some anti-inflammatory, antiviral, anticancer, and neuroprotective activities through different mechanisms of action [12]. One sub-group of flavonoid compounds is referred to as proanthocyanidins, because an acid-catalyzed cleavage of their polymeric chains generates anthocyanidins [13]. A recent study by Li et al. [14] conducted in a rat model of ulcerative colitis induced by 2,4,6-trinitrobenzene sulfonic acid (TNBS) revealed that proanthocyanidins from grape seeds exert a wide range of positive effects. These effects include downregulating some mediators involved in the intestinal inflammatory response, inhibiting inflammatory cell infiltration and oxidation damage, promoting damaged tissue repair, decreasing the production of proinflammatory interleukin IL-1β, and increasing the production of anti-inflammatory interleukins IL-2 and IL-4 [14].

Considering the potential positive influence of proanthocyanidins for treating ulcerative colitis, based on animal model studies, the present study aimed to verify the influence of dietary proanthocyanidins intake on the symptoms of ulcerative colitis in remission in human subjects.

## 2. Experimental Section

### 2.1. Study Design

The presented study was conducted in cooperation between the Dietetic Outpatient Clinic of the Institute of Human Nutrition Sciences, Warsaw University of Life Sciences (WULS-SGGW) with three gastroenterology outpatient clinics that consult patients with ulcerative colitis: (1) the Gastroenterology Outpatient Clinic of the Maria Skłodowska-Curie Memorial Cancer Center and Institute of Oncology, (2) the Gastroenterology Outpatient Clinic of the Central Clinical Hospital of the Ministry of Interior in Warsaw, and (3) the Gastroenterology Outpatient Clinic of the Public Central Teaching Hospital in Warsaw.

The study was conducted based on the approvals of following ethical committees: (1) Bioethical Commission of the Central Clinical Hospital of the Ministry of Interior in Warsaw (No. 35/2009), and (2) Bioethical Commission of the National Food and Nutrition Institute (No. 1604/2009). It was conducted according to the guidelines laid down in the Declaration of Helsinki, while participants provided their written informed consent to participate in the study.

### 2.2. Study Participants

The study participants were ulcerative colitis patients with confirmed remission from the following gastroenterology outpatient clinics: (1) the Gastroenterology Outpatient Clinic of the Maria Skłodowska-Curie Memorial Cancer Center and Institute of Oncology, (2) the Gastroenterology Outpatient Clinic of the Central Clinical Hospital of the Ministry of Interior in Warsaw, and (3) the Gastroenterology Outpatient Clinic of the Public Central Teaching Hospital in Warsaw.

They were recruited by their gastroenterologists, based on the following inclusion criteria:

- Being Caucasian;
- Being aged 18–80 years;
- Being nonhospitalized for any disease;
- Endoscopically diagnosed ulcerative colitis confirmed in the clinic records;
- Having confirmed remission of ulcerative colitis—endoscopic remission: image without any changes or disappearance of vascular network, erythema, inflammatory polyps allowed; clinical remission: assessed based on the Mayo Scoring System (the cut-off being two points in a 12-points scale); and the Rachmilewitz index (the cut-off being four points in a 31-points scale) for the assessment of the ulcerative colitis activity [15], as described previously [16];
- Having been in clinical remission for at least six weeks;
- Having had constant drugs doses for at least six weeks confirmed by patient

And the following exclusion criteria:

- Pregnancy;
- Diagnosed cancer;
- Any gastrointestinal resection.

Based on the indicated inclusion and exclusion criteria, the total number of 55 participants were recruited, while 19 males and 36 females volunteered to participate in the study. The characteristics of the studied group were presented in the previous publications, while the majority of respondents declared no more than three bowel movements per day in the period of remission [17], and the concurrent diseases did not differ between male and female respondents [18]. The demographic and clinical characteristics of the included patients are presented in Table 1.

### 2.3. Study Procedures

The study included the analysis of diet, as well as the analysis of the disease symptoms in the period of remission. The previous analysis verified the association between dietary intake of carotenoids [16,20], as well as isoflavones [17,21], and the symptoms of ulcerative colitis. However, those studies did not explain fully the influence of diet, so it was hypothesized, that there may be some other diet-related factor which may influence the disease. As proanthocyanidins are supposed to have been such a diet-related factor, based on the study by Wang et al. [22], it was assumed that potential positive influence of this component was to be verified.

#### 2.3.1. Disease Symptoms

The assessment of disease symptoms was based on the self-reported data, that were obtained from the respondents. They were asked about the following issues: abdominal pain, presence of blood, mucus and pus in their stool, constipations, flatulence, tenesmus, and daily number of bowel movements. In spite of the fact that all included patients were in confirmed remission, the applied criteria of the Mayo Scoring system and the Rachmilewitz index allowed the presented symptoms to be observed [15]. Moreover, such a situation, that some symptoms of inflammation are observed even in confirmed remission is stated to be quite common [23]. Taking this into account, participants may have been clustered based on the declared symptoms.

For the daily number of bowel movements, participants were asked about their typical number of bowel movements per day, on the typical day during their remission. They were asked to not focus only on the present day, or present week, but to declare the typical number for their general remissions.

For the abdominal pain, presence of blood, mucus and pus in their stool, constipations, flatulence, and tenesmus, participants were asked about each symptom separately, if they declared it during their remissions, while using the structured questionnaire. Before asking any question, participants were instructed that they should base their declarations only on their own feelings and observations, not on any suggestion, or supposition that they should have any symptoms, or on the results of their medical examinations. Patients did not have to describe precisely the intensity of their symptoms (e.g., intensity of abdominal pain), or amount (e.g., amount of blood in their stool), but they were only asked if such a symptom is for them present. The included symptoms of ulcerative colitis were based on the Qualitative Focus Group Study by Waljee et al. [24], as they identified which symptoms of ulcerative colitis are in fact important for patients, and they compared these symptoms with commonly measured ones to determine whether the patient-reported important symptoms are represented in current disease activity indices for ulcerative colitis. In the referred study, among other symptoms which are reported by patients, as recognized by them, but at the same time, which are not included to used indices of disease activity, there were: presence of mucus in stool, constipations and flatulence [24]. For the above-mentioned symptoms, Waljee et al. [24] indicated that participants of their study spent significant time in discussing the importance of anxiety concerning these symptoms and their influence on quality of life. Those symptoms may be indicated as not only important for patients and recognizable by them, but also as commonly neglected in the assessment of ulcerative colitis, which results in assessment of ulcerative colitis not being patient-centered and not associated with good understanding of the patient’s disease experience.

At the beginning patients were asked about specific symptoms—if they require to have a description of them presented and, if required, an image of them was verbally described. Afterwards, they were asked about their symptoms before the ulcerative colitis diagnosis and then, about the current symptoms (while ulcerative colitis has been diagnosed, but specifically it is in remission).

Finally, they were asked to summarize their observations, while specifying if the intensity or frequency of a symptom is during their remissions higher than it was previously (when they did not have ulcerative colitis). Based on the obtained data, participants were for each symptom divided into two subgroups: respondents declaring the symptom (both those who do not declare it for the period before ulcerative colitis, or declare that at present it is more intensive or more frequent), and respondents not declaring the symptom (to this group were included also those respondents who at present declare the symptom, but with the intensity and frequency comparable as for the period before ulcerative colitis).

#### 2.3.2. Dietary Intake

The habitual intake was assessed with no specific dietary intervention conducted. The dietary intake was assessed based on the three-day dietary records that were conducted by the participants during three typical, random, but not consecutive days, two of them being week days and one a weekend day [25]. Participants received structured forms with the detailed instructions, and the self-reported data were gathered. They were asked to record all the food products consumed, including beverages, while they had to describe the dishes with their ingredients, proportions and applied thermal treatment. To declare serving size, participants were allowed either to declare the mass of the consumed product (if they possessed a kitchen scale, or consumed packed product with the information about mass specified), or to describe the consumed serving using typical household measures.

Participants were asked for scrupulous recording of the products consumed and for not changing their typical dietary habits, due to recording. Afterwards, each participant had to describe orally the consumed dishes, while the dietitian asked additional questions, if it was necessary to specify any information. While describing the food, respondents were shown photographs of food products and dishes in a Polish atlas of food products’ serving sizes, to verify the serving sizes, and all participants had their three-day dietary records verified by the same dietitian, to reduce bias.

In order to assess the proanthocyanidins intake, the United States Department of Agriculture (USDA) Database for Proanthocyanidins Content of Selected Foods [26] was applied, as there is no Polish database for proanthocyanidins. At the same time, the energy value of diets was assessed to estimate also the proanthocyanidins intake recalculated per 1000 kcal of diet and to verify it as a potential interfering factor (energy recalculated as kcal per kg of body mass). The intake of other potential nutritional interfering factors was also calculated for the following: protein (recalculated as grams per kg of body mass and % of energy), fat (recalculated as % of energy), carbohydrates (recalculated as % of energy), sucrose (recalculated as % of energy), fiber, sodium, potassium, calcium, phosphorus, magnesium, iron, zinc, copper, vitamins A, D, E, B_1_, B_2_, B_6_, B_12_, folate, niacin, C. The Dietetyk 2.0, the Polish dietitian software with the nutritional value database [27], was applied.

#### 2.3.3. Applied Treatment as a Potential Interfering Factor

The applied treatment was taken into account as a potential interfering factor for the association between dietary intake and symptoms, so it was verified whether the applied treatment is associated with the proanthocyanidins intake. Patients were divided into groups stratified by: number of groups of applied medications for the groups of 5-aminosalicylic acid, corticosteroid and immunosuppressive medications (1 group/2 groups/3 groups), 5-aminosalicylic acid (applied/not applied), corticosteroid (applied/not applied), and immunosuppressive medications (applied/not applied). The conducted statistical analysis revealed that there is no such association (*p* > 0.05, U Mann–Whitney test).

### 2.4. Statistical Analysis 

The distribution was verified using the Shapiro–Wilk test. Afterwards, three-stage statistical analysis was conducted. Due to nonparametric distribution, in the first stage, the Mann–Whitney U test was applied to compare sub-groups characterized by various symptoms, for the proanthocyanidins intake. In the second stage it was applied to compare sub-groups for the energy intake and intake of other nutrients that may have interfered the observed association (protein, fat, carbohydrates, sucrose, fiber, sodium, potassium, calcium, phosphorus, magnesium, iron, zinc, copper, vitamins A, D, E, B_1_, B_2_, B_6_, B_12_, folate, niacin, C). Finally, in the third stage, in the cases when not only proanthocyanidins intake was stated to be associated with symptoms, but it was also observed for the other nutrients, the logistic regression was applied. Additionally, for daily number of bowel movements, the Spearman rank correlation coefficient was used to verify correlations.

The level of *p* ≤ 0.05 was accepted for the level of significance. All the statistical analyses were conducted using Statistica software 8.0 (StatSoft Inc., Tulsa, OK, USA) and PQStat software 1.6.6. (PQStat Software SBO, Plewiska, Poland).

## 3. Results

The comparison of proanthocyanidins intake between patients with and with no abdominal pain is presented in Table 2. It was observed that there is no difference of proanthocyanidins intake between patients with and with no abdominal pain, both for their daily intake (*p* = 0.4431) and intake recalculated per 1000 kcal of diet (*p* = 0.4040). As no difference of proanthocyanidins intake between patients with and with no abdominal pain was observed, the other stages of the statistical analysis were not conducted.

The comparison of proanthocyanidins intake between patients with and with no presence of blood in stool is presented in Table 3. It was observed that there is no difference of proanthocyanidins intake between patients with and with no presence of blood in stool, both for their daily intake (*p* = 0.4021) and intake recalculated per 1000 kcal of diet (*p* = 0.1451). As no difference of proanthocyanidins intake between patients with and with no presence of blood in stool was observed, the other stages of the statistical analysis were not conducted.

The comparison of proanthocyanidins intake between patients with and with no presence of mucus in stool is presented in Table 4. It was observed, that participants declaring presence of mucus in their stool compared with other participants were characterized by higher proanthocyanidins intake (142 vs. 75 mg; *p* = 0.0441) and intake per 1000 kcal (91 vs. 37 mg/1000 kcal; *p* = 0.0092). As there was a difference of proanthocyanidins intake between patients with and with no presence of mucus in stool, the other stages of the statistical analysis were conducted. The identical comparisons between patients with and with no presence of mucus in stool were conducted for energy value of diet and all possible interfering nutrients (protein, fat, carbohydrates, sucrose, fiber, sodium, potassium, calcium, phosphorus, magnesium, iron, zinc, copper, vitamins A, D, E, B_1_, B_2_, B_6_, B_12_, folate, niacin, C), but for none of them the difference of intake between patients with and with no presence of mucus in stool was stated (*p* > 0.05, U Mann–Whitney test).

The comparison of proanthocyanidins intake between patients with and with no presence of pus in stool is presented in Table 5. It was observed that there is no difference of proanthocyanidins intake between patients with and with no presence of pus in stool, both for their daily intake (*p* = 0.2010) and intake recalculated per 1000 kcal of diet (*p* = 0.2284). As no difference of proanthocyanidins intake between patients with and with no presence of pus in stool was observed, the other stages of the statistical analysis were not conducted.

The comparison of proanthocyanidins intake between patients with and with no constipations is presented in Table 6. It was observed, that participants declaring constipations while compared with other participants were characterized by higher proanthocyanidins intake (214 vs. 82 mg; *p* = 0.0289) and intake per 1000 kcal (118 vs. 41 mg/1000 kcal; *p* = 0.0194). As there was a difference of proanthocyanidins intake between patients with and with no constipations, the other stages of the statistical analysis were conducted. The identical comparisons between patients with and with no constipations were conducted for energy value of diet and all possible interfering nutrients (protein, fat, carbohydrates, sucrose, fiber, sodium, potassium, calcium, phosphorus, magnesium, iron, zinc, copper, vitamin A, D, E, B_1_, B_2_, B_6_, B_12_, folate, niacin, C). The statistically significant differences were stated for energy value of diet (*p* = 0.0350, U Mann–Whitney test) and protein intake recalculated as grams per kg of body mass (*p* = 0.0238, U Mann–Whitney test), as the higher energy value of diet and the higher protein intake were stated for participants not declaring constipations (median of 29.1 kcal/kg differing from 7.9 to 74.3 kcal/kg; median of 1.15 g/kg differing from 0.42 to 2.24 g/kg) than those who declared (median of 20.8 kcal/kg differing from 12.5 to 28.3 kcal/kg; median of 0.83 g/kg differing from 0.49 to 1.61 g/kg).

For all the nutritional factors that were stated to be associated with constipations incidence (daily proanthocyanidins intake, energy value of diet, protein intake), the logistic regression was conducted. It was revealed, that in the logistic regression model, while three indicated variables were taken into account, only daily proanthocyanidins intake remained a factor significantly associated with constipations incidence (*p* = 0.0183; OR = 1.01; 95% CI 1.00–1.02).

The comparison of proanthocyanidins intake between patients with and with no flatulence is presented in Table 7. It was observed that there is no difference of proanthocyanidins intake between patients with and with no flatulence, both for their daily intake (*p* = 0.8200) and intake recalculated per 1000 kcal of diet (*p* = 0.8399). As no difference of proanthocyanidins intake between patients with and with no flatulence was observed, the other stages of the statistical analysis were not conducted.

The comparison of proanthocyanidins intake between patients with and with no tenesmus is presented in Table 8. It was observed that there is no difference of proanthocyanidins intake between patients with and with no tenesmus, both for their daily intake (*p* = 0.3568) and intake recalculated per 1000 kcal of diet (*p* = 0.3811). As no difference of proanthocyanidins intake between patients with and with no tenesmus was observed, the other stages of the statistical analysis were not conducted.

The analysis of correlation between proanthocyanidins intake and daily number of bowel movements is presented in Table 9. It was observed that there is no correlation, both for daily intake (*p* = 0.5597; R = –0.0804) and intake recalculated per 1000 kcal of diet (*p* = 0.7695; R = –0.0404).

## 4. Discussion

Although a positive influence of proanthocyanidins from grape seeds on the course of ulcerative colitis in a rat model has been observed [14], the mechanism by which proanthocyanidins exert this effect remains unclear. Proanthocyanidins are found to be poorly absorbed in the proximal part of the gut, while the increased excretion of aromatic acids in rats fed with proanthocyanidins allow to hypothesize that proanthocyanidins possibly influence microbial activity after their absorption in the gastrointestinal tract [28]. However, based on the studies conducted so far by other authors, it must be specified that possibility to absorb proanthocyanidins, especially in the case of ulcerative colitis patients, is still unknown. Based on the current state of knowledge, proanthocyanidins cannot be defined as potentially absorbable, as it was not verified for this group of patients. We may attempt to specify the influence of proanthocyanidins, as was observed by other authors, but we must be aware that the mechanisms are unknown and may only be supposed.

A previous study by Li et al. [29] showed that proanthocyanidins from grape seeds exerted beneficial effects in a rat model of ulcerative colitis by inhibiting Nuclear Factor-Kappa B (NF-κB) signal transduction pathways. Similarly, a cell culture study found that the anti-inflammatory properties of proanthocyanidin-rich red rice extract were associated with the inhibition of proinflammatory mediators through the suppression of the NF-κB, Activator Protein-1 (AP-1), and Mitogen-Activated Protein Kinase (MAPK) pathways [30]. Proanthocyanidins from grape seeds also reduced the expression levels of Tumor Necrosis Factor-alpha (TNF-α), phosphorylated Inhibitor Kappa B Kinase (p-IKKα/β), and phosphorylated Inhibitor Kappa B-alpha (p-IκBα) as well as inhibited the translocation of NF-κB in the colonic mucosa, and thus exerted a protective effect on a rat recurrent-colitis model by modifying the inflammatory response and promoting damaged tissue repair to improve colonic oxidative stress [31]. The cytoprotective effects of proanthocyanidins from grape seeds were also observed independently of its antioxidant potential in another cell culture study [32].

While summarizing the effect of proanthocyanidins on the gastrointestinal tract, Cires et al. [33] stated that they may influence the gastrointestinal system and induce specific effects on: mouth (binding to salivary proteins, sensation of astringency, and inhibition of *Streptococcus mutans* adhesion); stomach (inhibition of *Helicobacter pylori* adhesion as well as its urease and Vacuolating cytotoxin A (VacA), antiemetic properties, slowing of gastric emptying, modulation of HCl and mucus secretion, and ulcer healing); small intestine (binding and precipitation of dietary proteins, binding and inhibition of enzymes, inhibition of nutrient transporters, interference with bile salts and micelle formation, modulation of secretion of enterohormones, prevention and reversion of diarrhea, and reduction of intestinal transit); and colon (fermentation and production of bacterial metabolites, modulation of intestinal microbiota, and protective effect against colorectal cancer).

Taking into account the observed results, it is crucial to properly interpret the effects of proanthocyanidins for colonic mucus and bowel movement frequency, as higher proanthocyanidins intake was noted for participants showing presence of mucus in stool and those with constipations.

Proanthocyanidins have the ability to stimulate mucus synthesis and secretion [34]. They also exhibit mucosal repair activity that is related to their antiulcer properties [33], as mucus may be beneficial for the gastrointestinal tract, especially for accelerating ulcer healing.

In spite of the fact that there are also factors other than ulcerative colitis that may influence mucus observed in stool, in a group of patients with confirmed disease, it may be above all interpreted as associated with this disease. Based on animal model studies, it may be also hypothesized that the improper mucosal intestinal layer may contribute to colitis development [35]. At the same time, the mucosal intestinal layer in ulcerative colitis patients in their exacerbation is thinner and more penetrable to fluorescent beads compared to healthy subjects [36]. It results from the fact that inflammatory process, typical for this disease, results in damage of intestinal mucosal layer and influences mucus excretion in stool [37], similarly as observed for other intestinal diseases [38]. Such situation is one of common symptoms of diseases in exacerbation [24], while the changes of composition and characteristics of mucus are also associated with the colonic content [39] and progression of the disease [40]. In spite of the fact that mucus layer degradation is typical for the phase of disease progression, it may be not only the effect of progression, but also it may influence further progression, as such degradation results in opening the intestinal barrier for microbial intestine aggression [41].

The pathway described above, associated with the ability of proanthocyanidins to stimulate mucus synthesis and secretion, may explain the results observed in the presented study, as individuals with the presence of mucus in stool showed a twice-higher intake of proanthocyanidins than other individuals. As the mechanism of the effect of proanthocyanidins, Iwasaki et al. [42] proposed that proanthocyanidins may inhibit the secretion of gastrin by G cells and also inhibit the secretion of histamine and somatostatin, in addition to increasing prostaglandin levels and superoxide dismutase activity in the mucosa and thereby preventing mucosal damage.

It is also indicated that proanthocyanidins may prevent or even revert diarrhea by decreasing fluid accumulation and reducing watery stools [43]. This pathway explains the results observed in the presented study, as individuals with constipations had more than twice-higher intake of proanthocyanidins than other individuals. Santos et al. [43] proposed that proanthocyanidins stimulate intestinal opioid receptors, without affecting intestinal motility, but at the same time, they may also delay carbohydrate digestion [44]. The effectiveness of proanthocyanidins to prevent diarrhea was even used in a patented pharmaceutical formulation of a proanthocyanidin polymer isolated from *Croton* spp. or *Calophyllum* spp. (Patent No. US 7341,744 B1) as a “Method of treating secretory diarrhea with enteric formulations of proanthocyanidins polymer” by Rozhon et al. [45].

On the basis of the results noted for the studied group, it could be considered that proanthocyanidins may have a beneficial effect on patients with ulcerative colitis. The antiulcer activity of mucus [33] may reduce the severity of disease, while constipation noted by patients may be associated with just lower frequency of bowel movements, which in the case of patients with ulcerative colitis is commonly elevated, even in remission [21].

In spite of the fact that the observations from the conducted study are interesting and may be promising, the limitations of the study must be also listed. The most important issue is associated with the fact that the studied group was homogenous (all participants in confirmed and constant remission), but as a result, it was relatively small. Moreover, in spite of the fact that numerous nutritional factors were taken into account, there may be also other potential food components that may influence the disease, such as other phenolic compounds, and other bioactive compounds. At the same time, also factors other than diet-related, e.g., associated with any concurrent diseases, age, or body mass may have influenced the observed symptoms. Last, but not least, the most reliable studies would be those assessing the effect of administration of a known amount and concentration of proanthocyanidins, or grape seed extract containing proanthocyanidins, in the controlled clinical trial. Such studies should be conducted in order to verify the observations from the presented analysis. However, it must be emphasized that it would be a model of supplementation, not of a dietary intake, as presented in presented study.

## 5. Conclusions

The positive influence of habitual dietary intake of proanthocyanidins was observed in the analysis conducted for the studied group of patients with ulcerative colitis in remission, so it may be supposed for such patients and should be verified in further studies. In the studied group, intake of proanthocyanidins may have increased the production of mucus, which may be beneficial for intestinal healing, and may have reduced the frequency of bowel movements. However, further experimental human studies are necessary to confirm the potential influence of proanthocyanidins intake in patients with ulcerative colitis in remission.

## Figures and Tables

**Table 1:** The demographic and clinical characteristics of included patients.

Characteristics	Mean ± SD	Median (minimum - maximum)	Share -*n* (%)	
**Gender**	–		Male	19 (34.5%)
			Female	36 (65.5%)
Age (years)	47.5 ± 13.8	49 (19–69)	–	
Body Mass Index (BMI) (kg/m^2^)	27.0 ± 5.2	26.3 (17.6–38.1)	Underweight	3 (5.4%)
			Normal weight	18 (32.7%)
			Overweight	19 (34.5%)
			Obesity	15 (27.3%)
Treatment duration (years)	6.69 ± 3.47	6 * (1–15)	–	
Hospitalizations (number/year)	0.35 ± 0.42	0.33 * (0–3)	–	
Number of groups of applied medications **	–		1 group	39 (70.9%)
			2 groups	11 (20.0%)
			3 groups	5 (9.1%)
Concurrent diseases ***	–		D50–D64	16 (29.1%)
			E00–E07	12 (21.8%)
			E10–E16	3 (5.4%)
			E78	3 (5.4%)
			F00–F99	7 (12.7%)
			G00–G99	3 (5.4%)
			I10–I15	14 (25.4%)
			J00–J99	4 (7.2%)
			K00–K46; K65–K93	28 (50.9%)
			L00–L99	8 (14.5%)
			M00–M99	20 (36.4%)
			N70–N99	3 (5.4%)

* nonparametric distribution (verified using the Shapiro–Wilk test; *p* ≤ 0.05); ** for the groups of 5-aminosalicylic acid, corticosteroid and immunosuppressive medications; *** based on International Statistical Classification of Diseases and Related Health Problems (ICD-10) [19], D50–D89: diseases of the blood and blood-forming organs, E00–E07: disorders of the thyroid gland, E10–E16: diabetes mellitus and other disorders of glucose regulation and pancreatic internal secretion, E78: disorders of lipoprotein metabolism and other lipidemias, F00–F99: mental and behavioral disorders, G00–G99: diseases of the nervous system, I10–I15: hypertensive diseases, J00–J99: diseases of the respiratory system, K00–K46, K65–K93: diseases of the digestive system other than noninfective enteritis and colitis as well as other diseases of the intestines, L00–L99: diseases of the skin and subcutaneous tissue, M00–M99: diseases of the musculoskeletal system and connective tissue, N70–N99: inflammatory diseases of female pelvic organs, as well as noninflammatory disorders of female genital tract and other disorders of the genitourinary system.

**Table 2:** Comparison of proanthocyanidins intake between patients with and with no abdominal pain.

Characteristics	Patients with Abdominal Pain (*n* = 26)	Patients with No Abdominal Pain (*n* = 29)	*p*-Value **
**Intake of proanthocyanidins (mg)**			
Mean ± SD	130.5 ± 105.3	119.9 ± 116.6	0.4431
Median (min-max)	102.1 * (7.4–374.5)	80.4 * (0.0–469.8)	
**Intake of proanthocyanidins (mg/1000 kcal)**			
Mean ± SD	77.3 ± 83.5	66.9 ± 88.6	0.4040
Median (min-max)	46.5 * (4.5–329.0)	42.2 * (0.0–462.4)	

* nonparametric distribution (verified using the Shapiro–Wilk test); ** compared using the U Mann–Whitney test (for non-parametric distributions).

**Table 3:** Comparison of proanthocyanidins intake between patients with and with no presence of blood in stool.

Characteristics	Patients with Presence of Blood in Stool (*n* = 38)	Patients with No Presence of Blood in Stool (*n* = 17)	*p*-Value **
**Intake of proanthocyanidins (mg)**			
Mean ± SD	135.9 ± 119.8	100.3 ± 84.1	0.4021
Median (min-max)	91.5 * (0.0–469.8)	77.2 * (0.0–337.7)	
**Intake of proanthocyanidins recalculated per 1000 kcal of diet (mg/1000 kcal)**			
Mean ± SD	83.9 ± 97.9	44.6 ± 37.6	0.1451
Median (min-max)	56.0 * (0.0–462.4)	38.7 * (0.0–160.0)	

* nonparametric distribution (verified using the Shapiro–Wilk test); ** compared using the U Mann–Whitney test (for non-parametric distributions).

**Table 4:** Comparison of proanthocyanidins intake between patients with and with no presence of mucus in stool.

Characteristics	Patients with Mucus in Stool (*n* = 9)	Patients with No Mucus in Stool (*n* = 46)	*p*-Value **
**Intake of proanthocyanidins (mg)**			
Mean ± SD	187.0 ± 130.0	112.8 ± 103.6	0.0441
Median (min-max)	141.8 * (86.5–86.5)	74.9 * (0.0–374.4)	
**Intake of proanthocyanidins recalculated per 1000 kcal of diet (mg/1000 kcal)**			
Mean ± SD	126.1 ± 130.0	61.1 ± 71.3	0.0092
Median (min-max)	90.8 * (41.3–462.4)	37.5 * (0.0–329.0)	

* nonparametric distribution (verified using the Shapiro–Wilk test); ** compared using the U Mann–Whitney test (for non-parametric distributions).

**Table 5:** Comparison of proanthocyanidins intake between patients with and with no presence of pus in stool.

Characteristics	Patients with Pus in Stool (*n* = 3)	Patients with No Pus in Stool (*n* = 52)	*p*-Value **
**Intake of proanthocyanidins (mg)**			
Mean ± SD	228.7 ± 209.2	118.9 ± 102.7	0.2010
Median (min-max)	122.1 (94.3–469.8)	85.0 * (0.0–374.4)	
**Intake of proanthocyanidins recalculated per 1000 kcal of diet (mg/1000 kcal)**			
Mean ± SD	187.4 ± 238.2	65.1 ± 68.6	0.2284
Median (min-max)	57.1 (42.7–462.4)	41.8 * (0.0–329.0)	

* nonparametric distribution (verified using the Shapiro–Wilk test); ** compared using the U Mann–Whitney test (for non-parametric distributions).

**Table 6:** Comparison of proanthocyanidins intake between patients with and with no constipations.

Characteristics	Patients with Constipations (*n* = 7)	Patients with No Constipations (*n* = 48)	*p*-Value **
**Intake of proanthocyanidins (mg)**			
Mean ± SD	240.2 ± 161.2	108.1 ± 91.9	0.0289
Median (min-max)	213.8 (32.0–469.8)	82.0 * (0.0–354.0)	
**Intake of proanthocyanidins recalculated per 1000 kcal of diet (mg/1000 kcal)**			
Mean ± SD	197.9 ± 172.3	53.4 ± 43.9	0.0194
Median (min-max)	118.1 (21.3–462.4)	41.2 * (0.0–179.2)	

* nonparametric distribution (verified using the Shapiro–Wilk test; *p* < 0.05); ** compared using the U Mann–Whitney test (for non-parametric distributions).

**Table 7:** Comparison of proanthocyanidins intake between patients with and with no flatulence.

Characteristics	Patients with Flatulence (*n* = 9)	Patients with No Flatulence (*n* = 46)	*p*-Value **
**Intake of proanthocyanidins (mg)**			
Mean ± SD	113.1 ± 67.5	127. ± 117.5	0.8200
Median (min-max)	120.3 (0.0–192.7)	85.0 * (0.0–469.8)	
**Intake of proanthocyanidins recalculated per 1000 kcal of diet (mg/1000 kcal)**			
Mean ± SD	53.7 ± 39.7	75.3 ± 91.8	0.8399
Median (min-max)	40.0 (0.0–115.5)	42.8 * (0.0–462.4)	

* nonparametric distribution (verified using the Shapiro–Wilk test); ** compared using the U Mann–Whitney test (for non-parametric distributions).

**Table 8:** Comparison of proanthocyanidins intake between patients with and with no tenesmus.

Characteristics	Patients with Tenesmus (*n* = 9)	Patients with no Tenesmus (*n* = 46)	*p*-Value **
**Intake of proanthocyanidins (mg)**			
Mean ± SD	87.5 ± 76.0	132.2 ± 115.3	0.3568
Median (min-max)	59.0 (0.0–212.5)	91.5 * (1.0–469.8)	
**Intake of proanthocyanidins recalculated per 1000 kcal of diet (mg/1000 kcal)**			
Mean ± SD	45.5 ± 38.5	76.9 ± 91.5	0.3811
Median (min-max)	31.4 (0.0–95.1)	42.8 * (0.9–462.4)	

* nonparametric distribution (verified using the Shapiro–Wilk test); ** compared using the U Mann–Whitney test (for non-parametric distributions).

**Table 9:** Analysis of correlation between proanthocyanidins intake and daily number of bowel movements.

Daily intake	*p*	R *
Proanthocyanidins (mg)	0.5597	–0.0804
Proanthocyanidins recalculated per 1000 kcal of diet (mg/1000 kcal)	0.7695	–0.0404

* Spearman rank correlation coefficient (for non-parametric distributions).

## Abbreviations

AP-1	Activator Proteins-1
MAPK	Mitogen-Activated Protein Kinase
NF-κB	Nuclear Factor Kappa B
p-IKKα/β	phosphorylated Inhibitor Kappa B Kinase
p-IκBα	phosphorylated Inhibitor Kappa B-alpha
TNBS	2,4,6-trinitrobenzene sulfonic acid
TNF-α	Tumor Necrosis Factor-alpha
USDA	United States Department of Agriculture
VacA	Vacuolating cytotoxin A
